# Reduced Growth of *Staphylococcus aureus* Under High Glucose Conditions Is Associated With Decreased Pentaglycine Expression

**DOI:** 10.3389/fmicb.2020.537290

**Published:** 2020-11-02

**Authors:** Zhen Luo, Shan Yue, Ti Chen, Pengfei She, Yuan Wu, Yong Wu

**Affiliations:** ^1^Department of Laboratory Medicine, The Third Xiangya Hospital, Central South University, Changsha, China; ^2^Department of Laboratory Medicine, Hunan Normal University School of Medicine, Changsha, China

**Keywords:** *Staphylococcus aureus*, glucose, eDNA, pentaglycine, lysostaphin resistance

## Abstract

The high-glucose-induced cytotoxicity in diabetes has been widely recognized. *Staphylococcus aureus* is the most frequent pathogen isolated from diabetic foot ulcers, but the properties of this bacterium under high glucose conditions remain unclear. *S. aureus* grew in medium usually forms weak biofilm, and which was significantly increased by addition of glucose. However, extracellular DNA (eDNA), an important component of biofilms, was markedly decreased in presence of 15 mM glucose. The reduced eDNA content was not caused by degradation, because the nuclease activity of biofilm supernatants with glucose was significantly decreased due to the acidic pH of the medium. Under planktonic state, the growth of *S. aureus* was significantly decreased in the Luria-Bertani (LB) medium supplemented with 25 mM glucose, and the reduced growth of *S. aureus* by glucose was dose-dependent. Except for glucose, the growth of planktonic *S. aureus* was also markedly decreased by fructose or sucrose. Amounts of acid metabolites were produced under high glucose conditions, but the survival of planktonic *S. aureus* was unaffected by these acidic conditions. Cells of *S. aureus* from the culture medium with glucose had a thinner cell wall and highly resistant to lysostaphin compared with the bacteria cultured in LB medium. mRNA expression of genes encoding pentaglycine bridges, the substrate of lysostaphin, was significantly decreased in *S. aureus* by glucose. In addition to *S. aureus*, the growth of *Staphylococcus haemolyticus* and *Staphylococcus epidermidis* was also significantly decreased by an excess of glucose, but strains of *Enterococcus faecalis*, *Escherichia coli*, and *Pseudomonas aeruginosa* were unaffected by glucose. In conclusion, the reduced growth of *S. aureus* under high glucose conditions is due to impairment of the unique cell-wall structure, pentaglycine bridges.

## Introduction

Glucose serves as an important carbohydrate for the growth of *Staphylococcus aureus*. Carbohydrates support the growth of *S. aureus* under anaerobic conditions and high nitric oxide stress (Richardson et al., 2008; Vitko et al., 2015). In the skin and soft-tissue infections model, glucose transporters contribute to the growth of *S. aureus*, which favors the fermentation of glucose over other carbohydrates (Vitko et al., 2016). Our previous study has revealed that fermentation of glucose contributes to aggregation of *S. aureus* (Luo et al., 2019). These data indicate that glucose is essential for the growth of *S. aureus*. However, high glucose usually induces endothelial and renal cytotoxicity in diabetic patients (Gao et al., 2015; Yi et al., 2018; Liu et al., 2020). *S. aureus* is the most frequent pathogen isolated from diabetic foot ulcers, but the properties of this bacterium under high glucose conditions remain unclear.

*Staphylococcus aureus* grew in medium usually forms weak biofilm, and addition of glucose to growth medium is a common practice to simulate biofilm formation *in vitro* (You et al., 2014). The matrix within biofilm often consists of polysaccharide intercellular adhesin (PIA). However, surface proteins, such as protein A, promote biofilm development in the absence of PIA exopolysaccharide (Merino et al., 2009). Protein A is a major component of the cell wall of *S. aureus* and is suppressed in tryptic soy broth (TSB) medium containing an excess of glucose (Yamada and Matsumoto, 1986). In addition, glucose enhances the killing efficiency of daptomycin against *S. aureus*, which is independent of the proton motive force and unaffected by physiological changes in pH (Prax et al., 2016). Daptomycin is a lipopeptide antimicrobial agent that hinders peptidoglycan synthesis in the bacterial cell wall. Daptomycin-resistant *S. aureus* are usually associated with thickening of the cell wall (Bertsche et al., 2011; Bayer et al., 2013). Therefore, glucose is known to be involved in cell-wall synthesis in *S. aureus*.

The major structural component of the cell wall of *S. aureus* is peptidoglycan, which is cross-linked by pentaglycine bridges that help maintain the integrity of the cell wall (Labischinski, 1992; Monteiro et al., 2019). Inhibition of pentaglycine bridge formation leads to a substantial decrease in peptidoglycan cross-links and renders the bacteria highly resistant to lysostaphin (Rohrer et al., 1999). Lysostaphin is a glycylglycine endopeptidase secreted by *Staphylococcus simulans*. Lysostaphin cleaves pentaglycine bridges in staphylococci, thereby hydrolyzing the cell wall and lysing the bacteria (Grundling and Schneewind, 2006; Francius et al., 2008). Several studies have demonstrated that *S. aureus* is susceptible to lysostaphin *in vitro* and *in vivo*, indicating that lysostaphin is a promising candidate for treating *S. aureus* infections (Wu et al., 2003; Oluola et al., 2007; Yang et al., 2007; Chen et al., 2014). However, a recent study reported that *S. aureus* was highly resistant to lysostaphin in nutrient-rich TSB medium (Wu et al., 2019). As glucose is the most important carbon source for *S. aureus*, we hypothesized whether glucose contributes to the lysostaphin resistance observed in *S. aureus* cultured in nutrient-rich medium. Lysostaphin-resistant strains usually exhibit reduced logarithmic growth rate and increased susceptibility to elevated temperatures (Kusuma et al., 2007). Therefore, we speculate that high glucose reduces the expression of pentaglycine, resulted in impaired growth of *S. aureus*.

## Materials and Methods

### Bacterial Strains and Culture Conditions

Eight clinical strains of *S. aureus* and one clinical strain of *Staphylococcus haemolyticus* (SH01) were isolated from patients with diabetic foot ulcers in the Department of Laboratory Medicine, Third Xiangya Hospital, Central South University, Hunan, China. All clinical isolates were identified by matrix-assisted laser desorption/ionization time-of-flight mass spectrometry (MALDI-TOF MS; Bruker Biotyper, Germany), and the identification scores were all above 2.0. *S. aureus* Newman was kindly provided by Professor Min Li (Department of Laboratory Medicine, Renji Hospital, Shanghai Jiao Tong University, China), *Staphylococcus epidermidis* RP62A was supplied by Professor Di Qu (Fudan University, Shanghai, China), and *Enterococcus faecalis* ATCC29212, *Escherichia coli* ATCC25922, and *Pseudomonas aeruginosa* ATCC27853 were kindly provided by Juncai Luo (Hunan Changsha Tiandiren Biotech Co., Ltd., Changsha, China). Bacteria were routinely cultured at 37°C on 5% sheep blood agar (BA) plates (Caring Trading Co., Ltd., Guangdong, China).

### CFU Assays

To determine the growth and survival of bacteria (*S. aureus*, *S. haemolyticus, S. epidermidis, E. faecalis, E. coli*, and *P. aeruginosa*) in the presence of sugar, overnight cultures grown in Luria-Bertani (LB) medium at 37°C were diluted 1:100 into fresh LB medium supplemented with 25 mM glucose (Sigma), 25 mM fructose (Sigma), or 12.5 mM sucrose (Sigma). Dose-dependent growth of *S. aureus* in glucose was assessed by culturing *S. aureus* Newman in fresh LB medium supplemented with 0, 5, 15, and 25 mM glucose. All cultures were incubated at 37°C with shaking at 180 rpm, and the colony-forming units (CFUs) were determined after 24 and/or 48 h by plating 5 μL serial dilutions on BA, incubating overnight at 37°C, and enumerating the number of colonies.

### Analysis of eDNA by Quantitative PCR

Extracellular DNA (eDNA) within biofilms was examined by using quantitative polymerase chain reaction (PCR) targeting the gene *gyrB* (Dengler et al., 2015). Briefly, overnight cultures of *S. aureus* Newman grown in LB medium at 37°C were diluted 1:100 into fresh LB medium containing 0, 1, 3, 5, or 15 mM glucose and 6 mL of these suspensions were cultured in 6-well flat bottom polystyrene plates (Corning, China) at 37°C for 18 h. The supernatants were carefully removed, and the biofilms were harvested, resuspended in 500 μL TE buffer, and mixed at 3000 rpm for 5 min with Mixmate (Eppendorf, Germany). A 10 μl aliquot was removed for bacterial numeration. After centrifugation at 12,000 *g* for 5 min, the supernatant was passed through a 0.22-μm pore-size filter (Millipore), then eDNA in the filtered supernatants was purified using a nucleic acid extraction kit (Da An Gene Co., Ltd., Sun Yat-sen University, China). The eDNA content was quantified by real-time PCR using the following conditions: pre-denaturation at 94°C for 2 min; 40 cycles of denaturation at 94°C for 15 s, annealing at 55°C for 20 s, and elongation at 72°C for 20 s. Primers used for PCR are shown in the Supplementary Table 1. The eDNA content was also examined with a commercialized PCR-based molecular methicillin-resistant *S. aureus* (MRSA) detection kit (Liferiver, China) according to manufacturer’s instructions.

### Nuclease Activity Assays

Overnight cultures of *S. aureus* Newman grown in LB medium at 37°C were diluted 1:100 into fresh LB medium with or without 15 mM glucose, and 6 mL of these suspensions were cultured in 6-well flat bottom polystyrene plates at 37°C for 18 h. The supernatants were collected, centrifuged at 3000 rpm for 10 min, the resulting supernatant was passed through a 0.22-μmpore-size filter, and the resulting filtrate was considered as biofilm supernatants. A culture was also established where the pH of the medium supplemented with glucose was adjusted to 7.5. For genomic DNA (gDNA) solution, overnight cultures of Newman were centrifuged at 12,000 *g* for 5 min and the resulting precipitate was washed twice with sterile normal saline, resuspended in TE buffer, and boiled at 100°C for 10 min. The suspension was centrifuged at 12,000 *g* for 15 min and the supernatant was considered as gDNA solution. Twenty microliters of gDNA solution was added to 180 μL biofilm supernatant and incubated at 37°C for 30 min. The remaining gDNA in the solution was quantified by real-time PCR as described above.

### Survival of *S. aureus* Newman Under Acidic Conditions

*Staphylococcus aureus* Newman was grown in LB medium overnight, then 3 mL of this culture was incubated in 15-mL tubes with 25 mM glucose, 20 mM lactic acid (Sigma), or 20 mM acetic acid (Sigma) for 48 h. CFUs of the cultures were determined by performing serial dilutions and plating 5 μL on BA or L-form induced agar plates (LIA, Caring Trading Co., Ltd., Guangdong, China). Plates were incubated overnight at 37°C before enumeration of colonies.

### Gram-Staining Analysis

A smear of bacterial culture (50 μL) was placed on a glass slide, thoroughly air dried, heat fixed, and then stained with a commercial Gram-staining solution (BASO Diagnostics, China). Briefly, the staining process comprised 10 s in crystal violet solution, 10 s in iodine solution, 20 s wash in decolorizer, then a final counter stain with safranin solution for 10 s. The stained slides were examined by an microscope (1000×).

### Transmission Electron Microscopy

Bacteria were collected by centrifugation at 3000 *g* for 10 min and were washed twice with phosphate-buffered saline (PBS). These bacteria were resuspended in 2.5% glutaraldehyde solution with Millonig’s phosphate buffer and shipped to the Transmission Electron Microscopy (TEM) Laboratory at the Pathology Department of Xiangya Hospital, Changsha, Hunan. Specimens were examined and photographed on a Hitachi HT-7700 electron microscope.

### Lysostaphin and Lysozyme Lysis Assay

Lysostaphin lysis assays were performed as previously reported with minor modifications (Grundling et al., 2006). Briefly, overnight cultures of *S. aureus, S. epidermidis*, or *S. haemolyticus* were diluted 1:100 into fresh LB medium with or without 25 mM glucose and were cultured at 37°C, 180 rpm for 24 h. For inhibition of glycolysis by 2-deoxy-D-glucose (2-DG), overnight cultures of *S. aureus* were diluted 1:100 into fresh LB medium containing 15 mM glucose and 60 mM 2-DG (Sigma) and were cultured at 37°C, 180 rpm for 8 h. These above cultures were then washed twice with PBS and resuspended to an OD_630_ of 1.8–2.1. This value was set as 100% at 0 min. Lysostaphin (Sigma) or lysozyme (Sigma) was added at final concentrations of 10 and 50 μg/mL, respectively. OD_630_ measurements were recorded at timed intervals, and data were plotted as percent OD_630_ values of the initial reading.

### Real-Time RT-PCR

Total RNA extraction was extracted from *S. aureus* Newman as described previously (Beltrame et al., 2015). Initially, cells were lysed using lysostaphin and Trizol (Sigma), then total RNA was extracted and purified using a nucleic acid extraction kit (Enzo Life Sciences) according to the manufacturer’s instructions. The concentration of the extracted RNA was evaluated by using a NanoDrop 1000 (Thermo Fisher Scientific). A total of 1 μg RNA was then reverse transcribed to cDNA using a reverse transcription kit (TransGen Biotech, Beijing, China) according to the manufacturer’s instructions. Briefly, real-time PCR was conducted under the following conditions: pre-denaturation at 94°C for 5 min; 40 cycles of denaturation at 94°C for 30 s, annealing at 57°C for 20 s, and extension at 72°C for 10 s. Relative expression was determined by using the 2^–ΔΔCt^ method. The level of transcription was determined relative to the expression of *gyrB*. Primers used for this real-time PCR were reported in a previous study (Supplementary Table 1; Brahma et al., 2019).

### Statistical Analysis

All data produced in this study was normal distributions, and an unpaired Student’s *t*-test was used to further evaluate the difference between two groups. All probabilities were two-tailed and *p* < 0.05 was considered as significant. Data were presented as the mean ± standard deviation (SD). Statistical analyses were performed by using GraphPad Prism version 5 (GraphPad Software, San Diego, CA).

## Results

### Reduced eDNA Release of *S. aureus* by Glucose

**FIGURE 1:**
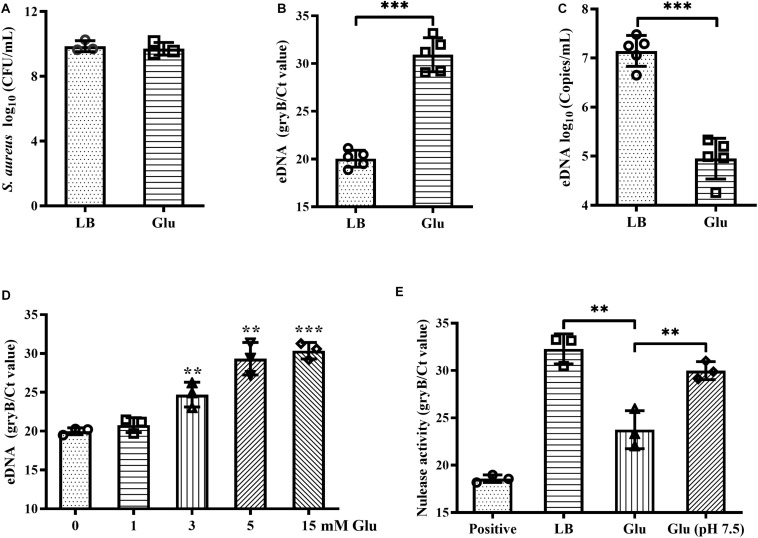
Glucose reduced eDNA levels of *S. aureus* biofilms. **(A)** CFUs of biofilms of *S. aureus* Newman cultured in LB medium with or without 15 mM glucose for 18 h. Supernatants were carefully removed, biofilms were harvested by resuspended in 500 μL TE buffer, and the CFUs of the biofilms were determined. **(B)** eDNA of biofilms quantified by real-time PCR targeting the gene *gyrB*. **(C)** eDNA of biofilms quantified using a commercialized PCR-based detection kit. **(D)** eDNA of biofilms of *S. aureus* Newman cultured in LB medium supplemented with 0, 1, 3, 5, or 15 mM glucose for 18 h. **(E)** Quantification of gDNA remaining after co-culture of gDNA solution with biofilm supernatants for 30 min; gDNA was quantified by real-time PCR targeting *gyrB*. ^∗∗^*p* < 0.01; ^∗∗∗^*p* < 0.001.

Addition of glucose to the growth medium simulates biofilm formation by *S. aureus* (Boles and Horswill, 2008). However, in this study, the CFUs of biofilms of *S. aureus* Newman were unaffected by addition of 15 mM glucose (Figure 1A). eDNA is an important component of biofilms, and which was markedly decreased by glucose (Figures 1B,C), concordant with results from a previous study (Sugimoto et al., 2018). Within biofilms, this reduction of eDNA by glucose was dose-dependent (Figure 1D). eDNA levels within biofilms are determined by eDNA release and degradation. Therefore, nuclease activity in the biofilm supernatants of *S. aureus* Newman was examined and was found to decrease significantly in the presence of glucose compared with LB medium (Figure 1E). Amount of acidic metabolites produced during the biofilm formation under high glucose condition may affect the nuclease activity of biofilm supernatants. After adjusting the pH of biofilm supernatants with glucose to 7.5, the nuclease activity was restored to almost levels observed in LB medium (Figure 1E). These data indirectly indicate that glucose reduces eDNA release but not eDNA degradation.

### Reduced Growth and Survival of *S. aureus* Under High Glucose Conditions

**FIGURE 2:**
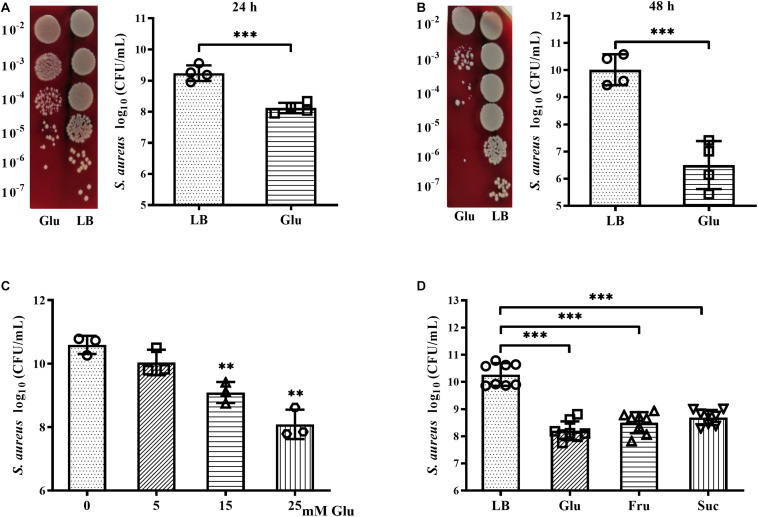
Glucose reduced growth and survival of *S. aureus* Newman. **(A)** CFUs of *S. aureus* Newman cultured in LB medium with or without 25 mM glucose for 24 h, with serial dilution and plating on BA. **(B)** CFUs of *S. aureus* Newman cultured in LB medium with or without 25 mM glucose for 48 h. **(C)** CFUs of *S. aureus* Newman cultured in LB medium supplemented with 0, 5, 15, or 25 mM glucose for 24 h. **(D)** CFUs of eight clinical *S. aureus* isolates from diabetic patients with foot ulcers cultured in LB medium with or without 25 mM glucose, 25 mM fructose, or 12.5 mM sucrose for 24 h. ^∗∗^*p* < 0.01; ^∗∗∗^*p* < 0.001.

The growth and survival of planktonic *S. aureus* Newman in the presence of glucose was examined. After culture for 24 h, CFUs of *S. aureus* Newman were reduced nearly 10-fold following addition of 25 mM glucose (Figure 2A). An increase in culture time to 48 h did not affect the CFUs of *S. aureus* Newman from the medium without glucose, but the CFUs of *S. aureus* Newman from the medium with glucose were further reduced by more than 1000-fold at this time point (Figures 2A,B). These data indicated that growth and survival of *S. aureus* Newman was significantly decreased by high concentrations of glucose. Next, the growth of *S. aureus* Newman in the presence of different concentrations of glucose was investigated. Reduced growth of this strain in the presence of glucose was observed to be dose-dependent (Figure 2C). Finally, the growth of eight clinical *S. aureus* isolates from patients with diabetic foot ulcers was examined in the presence and absence of glucose, and a high concentration of glucose (25 mM) was found to significantly reduce growth of these clinical isolates after culture for 24 h (Figure 2D). Similar results were observed following addition of fructose or sucrose (Figure 2D). These data indicate that the growth and survival of *S. aureus* is impaired under high glucose conditions.

### Survival of *S. aureus* Under Acidic Conditions

**FIGURE 3:**
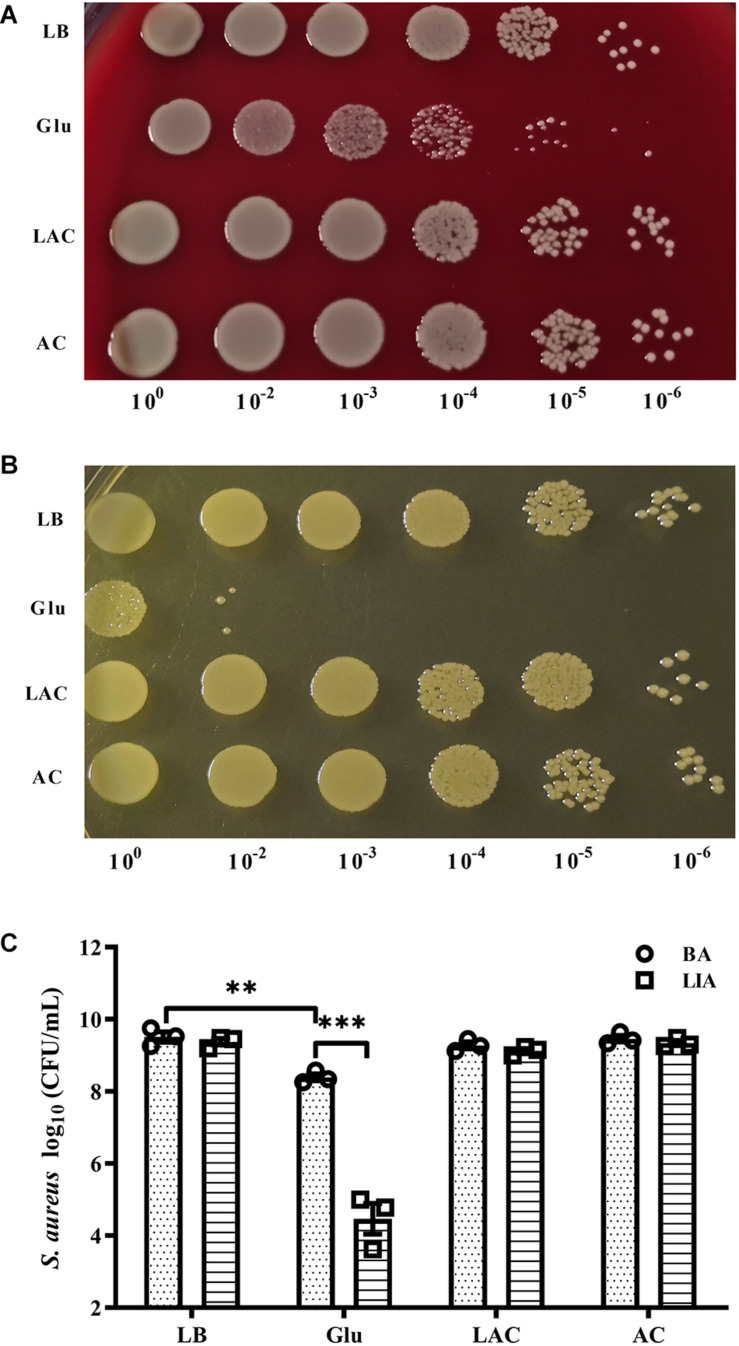
Survival of *S. aureus* Newman was unaffected by organic acid. *S. aureus* Newman was grown overnight in LB medium, then 25 mM glucose, 20 mM lactic acid, or 20 mM acetic acid was added to the medium and the cultures were incubated for a further 48 h. CFUs were then determined by serial dilution and plating on BA **(A,C)** and LIA plates **(B,C)**. ^∗∗^*p* < 0.01; ^∗∗∗^*p* < 0.001.

The pH of growth medium of *S. aureus* Newman in the planktonic state dropped to 5.0 after culture for 8 h in the presence of 25 mM glucose (data not shown). To determine whether acidic conditions affected survival of *S. aureus*, 20 mM exogenous lactic acid or acetic acid was added to overnight cultures of *S. aureus* Newman, resulting in the culture medium reaching a pH of 5.0 (data not shown). After culture for 48 h under these acidic conditions, the bacteria were serially diluted and plated on BA and LIA plates. On the BA plates, CFUs of *S. aureus* Newman significantly decreased following addition of 25 mM glucose, but were unchanged following addition of exogenous lactic acid or acetic acid (Figures 3A,C). Similar results were observed on LIA plates (Figures 3B,C). These data indicate that survival of *S. aureus* Newman under high glucose conditions is unaffected by acidic metabolites.

### Reduced Cell-Wall Thickness of *S. aureus* Under High Glucose Conditions

**FIGURE 4:**
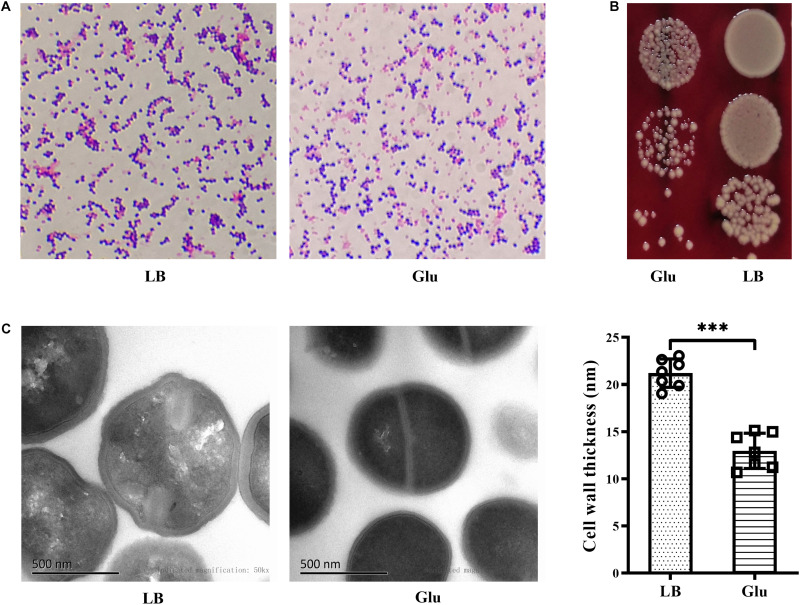
Glucose reduced the thickness of the cell wall of *S. aureus* Newman. *S. aureus* Newman was cultured in LB medium with or without 25 mM glucose. **(A)** After cultured for 48 h, Gram-staining was used to examine morphology changes of *S. aureus* by glucose. **(B)** After 24 h, culture medium were diluted and plated on BA. **(C)** After 48 h, bacterial cells were analyzed by TEM, and the thickness of the cell wall was measured. Graphs show representative results from two independent experiments. ^∗∗∗^*p* < 0.001.

Gram-positive bacteria are known to have a thick cell wall, whereas Gram-negative bacteria have a thin cell wall. Changes in the morphology of *S. aureus* in the presence of glucose were firstly investigated using Gram-staining. *S. aureus* is Gram-positive, but the proportion of Gram-stain-negative bacteria from the culture medium with glucose was markedly increased compared to those from LB medium (Figure 4A). To exclude the possibility of Gram-negative bacteria as contaminants, the culture medium were diluted, plated on BA, and incubated overnight. Only different-sized colonies of *S. aureus* grew on BA after overnight culture (Figure 4B). TEM analysis was used to further determine structural changes in the cell wall of *S. aureus* Newman in the presence of glucose. As shown in Figure 4C, the thickness of the bacterial cell wall was significantly decreased by a high concentration of glucose. These data indicate that *S. aureus* displays defects in the cell wall under high glucose conditions.

### Reduced Pentaglycine Expression of *S. aureus* Under High Glucose Conditions

**FIGURE 5:**
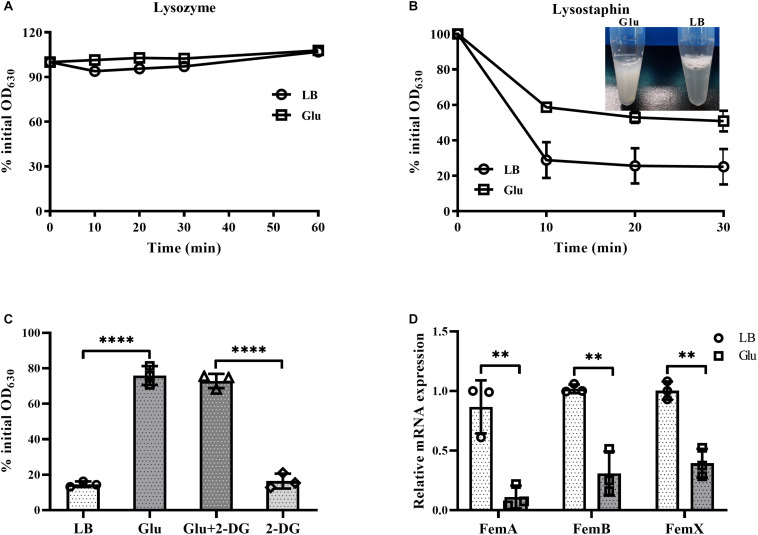
Glucose reduced expression of pentaglycine. *S. aureus* Newman was cultured in LB medium with or without 15 mM glucose for 24 h. Bacterial cells were collected by centrifugation and resuspended in PBS buffer, with lysozyme **(A)** or lysostaphin **(B)** added at a final concentration of 50 and 10 μg/mL, respectively. OD_630_ measurements were taken at timed intervals. **(C)** Lysostaphin resistance of *S. aureus* Newman in medium supplemented with 15 mM glucose was unaffected by the addition of 60 mM 2-DG. **(D)** *S. aureus* Newman was cultured in LB medium with or without 15 mM glucose for 6 h, and bacterial cells were collected for analysis of mRNA expression of *femA*, *femB*, and *femX*. ^∗∗^*p* < 0.01; ^****^p < 0.0001.

*Staphylococcus aureus* is completely resistant to the hydrolytic activity of lysozyme (Bera et al., 2007). This resistance of *S. aureus* to lysozyme was unaffected by the addition of glucose (Figure 5A). In contrast, *S. aureus* is susceptible to the enzyme lysostaphin, which recognizes the pentaglycine bridges between peptidoglycan chains, and then drives the preferential digestion of cell walls (Grundling and Schneewind, 2006). However, cells of *S. aureus* from the culture medium with glucose were highly resistant to lysostaphin compared to those from LB medium (Figure 5B). In addition, lysostaphin resistance induced by glucose was unaffected by the inhibitor of glycolysis, 2-DG (Figure 5C). These data suggest that the expression of pentaglycine bridges in *S. aureus* may be affected by glucose. Members of the factor essential for methicillin resistance ABX (*femABX*) family are required for synthesis of pentaglycine crossbridges in staphylococci (Monteiro et al., 2019). Therefore, expression of *femA*, *femB*, and *femX* mRNA was examined in the cultures of *S. aureus*. As shown in Figure 5D, mRNA levels of *femA*, *femB*, and *femX* were significantly decreased in the presence of glucose. These data indicate that high glucose reduces the synthesis of pentaglycine bridges between peptidoglycan chains in *S. aureus*.

### The Unique Cell-Wall Structure Is Associated With the Reduced Growth of Staphylococci by Glucose

**FIGURE 6:**
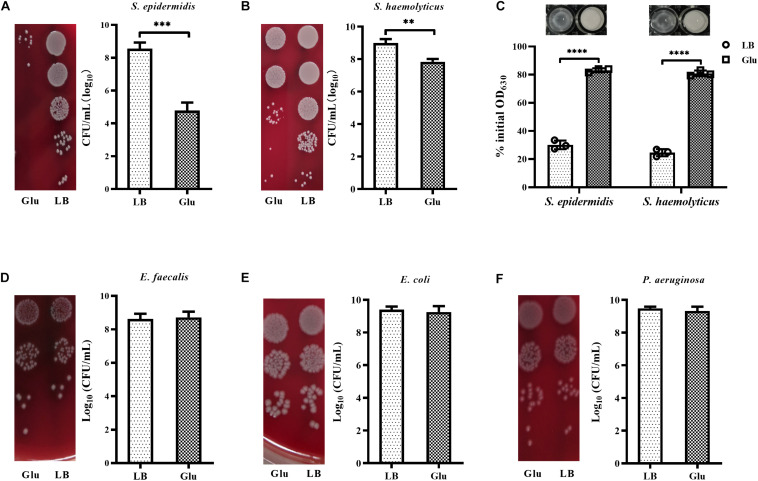
Glucose reduced the growth of staphylococci. **(A)** *S. epidermidis* RP62A and **(B)** *S. haemolyticus* SH01 were cultured in LB medium with or without 25 mM glucose for 48 h, then the CFUs of the cultures were determined by serial dilution and plating on BA. **(C)** *S. epidermidis* RP62A and *S. haemolyticus* SH01 were cultured in LB medium with or without 25 mM glucose for 24 h. The bacterial cells were collected by centrifugation, resuspended in PBS buffer, and lysostaphin was added at a final concentration of 10 μg/mL. OD_630_ measurements were recorded after 30 min. **(D)** *Enterococcus faecalis* ATCC29212, **(E)** *Escherichia coli* ATCC25922, and **(F)** *P. aeruginosa* ATCC27853 were cultured in LB medium with or without 25 mM glucose for 48 h, then CFUs of the cultures were determined by serial dilution and plating on BA. ^∗∗^*p* < 0.01; ^∗∗∗^*p* < 0.001; ^****^p < 0.0001.

Peptidoglycan is the main component of bacterial cell walls, the composition of which varies considerably between species. Staphylococci possess highly cross-linked peptidoglycan due to the presence of pentaglycine bridges. To investigate whether glucose affected the growth of other staphylococci. In addition to *S. aureus*, the growth of *S. epidermidis* and *S. haemolyticus* in the presence of 25 mM glucose was examined. Addition of glucose significantly reduced the growth of *S. epidermidis* and *S. haemolyticus* (Figures 6A,B). Furthermore, *S. epidermidis* and *S. haemolyticus* were highly resistant to lysostaphin in the LB medium with 25 mM glucose (Figure 6C). However, growth of *E. faecalis* and Gram-negative bacteria, such as *E. coli* and *P. aeruginosa*, was unaffected by glucose (Figures 6D–F). These data indicate that the unique cell-wall structure of staphylococci results in their reduced growth in the presence of glucose.

## Discussion

High-glucose-induced cytotoxicity has been observed in various eukaryotic cells, such as endothelial cells and fibroblasts (Gao et al., 2015; Sedlic et al., 2017; Buranasin et al., 2018). In this study, a high concentration of glucose was found to significantly reduce growth and survival of planktonic *S. aureus*. A previous study reported that growth of *E. coli* in unbuffered LB medium was markedly decreased by the addition of 11.11 mM glucose (Kwiatkowska et al., 2008). The pH of the growth medium dropped to 5.0 in the presence of 25 mM glucose in this study and our previous study (Luo et al., 2019). These data suggested that acidic metabolites might contribute to the reduced growth and survival of *S. aureus* by glucose. The lactic acid content of the culture medium during aggregation of *S. aureus* induced by glucose was less than 15 mM (Luo et al., 2019), therefore 20 mM lactic acid or acetic acid was added into the overnight cultures of *S. aureus*, resulting in the pH of the cultures decreasing to 5.0. Survival of *S. aureus* during stationary phase was unaffected by these acidic conditions, indicating that the reduced growth and survival of *S. aureus* under high glucose conditions is not due to the presence of acidic metabolites. Contrary to these data, murine footpad infection models revealed that hyperglycemia favors bacterial survival (Tuchscherr et al., 2018). Therefore, other internal and external factors, such as stain lineage and serum components, also affect the growth and survival of strains of *S. aureus* (Croes et al., 2009; Yin et al., 2017).

*Staphylococcus aureus* forms robust biofilms in growth medium supplemented with glucose, and the increased biofilm formation induced by glucose has a threshold response at clinically important concentrations (Waldrop et al., 2014). During biofilm formation, a population of cells release extracellular matrix, such as extracellular proteins and eDNA, to form multicellular clusters. The eDNA component within biofilms is released into the supernatant when the biofilms are suspended in a buffer with a higher pH (Dengler et al., 2015); this enables eDNA within biofilms to be experimentally separated from gDNA. Herein, glucose (>3 mM) significantly reduced eDNA levels within biofilms, and the amount of glucose was less than that reported in a previous study (Sugimoto et al., 2018). A previous study demonstrated that 22.22 mM glucose could repress nuclease transcription and prevent nuclease accumulation (Kiedrowski et al., 2011). In our study, nuclease activity of biofilm supernatants was significantly reduced in the presence of 15 mM glucose compared with LB medium, and this activity could be restored almost to levels recorded in LB medium by adjusting the pH of the culture solution to 7.5. Therefore, eDNA does not contribute to the increase in biofilm formation induced by glucose; other factors are involved in this process.

In addition to *S. aureus*, growth of strains of *S. epidermidis* and *S. haemolyticus* was also markedly decreased by glucose, while growth of *E. faecalis*, *E. coli*, and *P. aeruginosa* was unaffected by glucose. Furthermore, like *S. aureus*, strains of *S. epidermidis* and *S. haemolyticus* were highly resistant to lysostaphin under high glucose conditions. Pentaglycine bridges, which are cleaved by lysostaphin, occur in the cell envelope of staphylococci but not in other bacterial species. Therefore, the unique cell-wall structure of staphylococci contributes to the reduced growth and survival of *S. aureus* in high glucose conditions.

The thickness of the cell wall of *S. aureus* was significantly reduced under high glucose conditions. Staphylococci possess highly cross-linked peptidoglycan due to the presence of the bridges containing five glycine molecules; these pentaglycine bridges are synthesized by the *femABX* family (Rohrer et al., 1999; Monteiro et al., 2019). Deletion of *femAB* genes reduces cross-linking between peptidoglycan chains, and results in bacterial strain being highly lysostaphin resistant (Stranden et al., 1997). Cells from the medium supplemented with glucose were highly resistant to lysostaphin. In addition, mRNA expression of genes of the *femABX* family in *S. aureus* Newman was significantly reduced by glucose compared with expression levels in LB medium. Therefore, culture of *S. aureus* under high glucose conditions leads to decreased pentaglycine expression in the bacteria and subsequent lysostaphin resistance.

Lysostaphin-resistant strains of *S. aureus* exhibit a reduced rate of exponential growth compared to lysostaphin-sensitive strains (Kusuma et al., 2007). Furthermore, cells of *S. aureus* do not survive without pentaglycine bridges, appearing as pseudomulticellular forms that eventually lyse due to extensive membrane rupturing (Monteiro et al., 2019). Our previous study reported that under high glucose conditions, cells of *S. aureus* formed multicellular arrangements and sank to the bottom of the culture vessel (Luo et al., 2019). Therefore, the reduced growth and survival of strains of *S. aureus* in high glucose is due to decreased expression of pentaglycine.

Lysostaphin shows rapid bactericidal activity against methicillin-susceptible and -resistant strains of *S. aureus*, with minimum inhibitory concentrations (MICs) ranging from 0.03 to 2 μg/mL, as determined using an agar dilution assay (Yang et al., 2007). Recent studies have reported that lysostaphin combined with other agents, such as LL-37 peptide, liposomal vancomycin, and nisin, are effective against *S. aureus* infection (Ceotto-Vigoder et al., 2016; Hajiahmadi et al., 2019; Sadeghi et al., 2019). However, the MIC values of lysostaphin are greatly increased by serial subculture (Gutierrez et al., 2020). Moreover, high levels of glucose and rich medium also lead to lysostaphin resistance (Wu et al., 2019). Therefore, utilization of lysostaphin to treat *S. aureus* infections should consider the factors affecting lysostaphin sensitivity.

## Data Availability Statement

The raw data supporting the conclusions of this article will be made available by the authors, without undue reservation, to any qualified researcher.

## Author Contributions

ZL, YuW, and YoW designed the study and analyzed the results. ZL, SY, TC, and PS conducted the experiments. ZL and YuW wrote the manuscript. SY, TC, PS, and YoW reviewed the manuscript. All authors contributed to the article and approved the submitted version.

## Conflict of Interest

The authors declare that the research was conducted in the absence of any commercial or financial relationships that could be construed as a potential conflict of interest.

## Abbreviations

GyrB: gyrase B
MIC: minimum inhibitory concentration
TEM: transmission electron microscopy
eDNA: extracellular DNA
gDNA: genomic DNA
CFUs: colony-forming units
PBS: phosphate-buffered saline
PIA: polysaccharide intercellular adhesin
TSB: tryptic soy broth
MALDI-TOF MS: matrix-assisted laser desorption/ionization time-of-flight mass spectrometry
BA: blood agar
LB: Luria-Bertani
2-DG: 2-deoxy-D-glucose
SD: standard deviation
*femABX*: factor essential for methicillin resistance ABX
OD: optical density
mRNA: messenger RNA
PCR: polymerase chain reaction
LIA: L-form induced agar.
