# Supplementary material for: Reduced Growth of Staphylococcus aureus Under High Glucose Conditions Is Associated With Decreased Pentaglycine Expression
Source: Front Microbiol. 2020 Nov 2;11:537290. doi: 10.3389/fmicb.2020.537290 (PMC7667020; doi:10.3389/fmicb.2020.537290)
Supplement: Supplementary file 1 [file Table_1.DOC]

**Supplement table 1. List of real-time PCR primers used in this study**

| Primer | Sequence (5’-3’) | Reference |
| --- | --- | --- |
| gyrB-forward | CAAATGATCACAGCATTTGGTACAG | (Brahma et al., 2019) |
| gyrB-reverse | CGGCATCAGTCATAATGACGAT |
| FemA-forward | AAATTGGGAGCAGCATCAGT | (Brahma et al., 2019) |
| FemA-reverse | GCAGCTGAATTCCCATTTTC |
| FemB-forward | TCGTGCCATTTGAAGGTCG | (Brahma et al., 2019) |
| FemA-reverse | TCAAGGTTTAATACGCCCATCC |
| FemX-forward | GCGAAGAATCGCTGTAGGTC | (Brahma et al., 2019) |
| FemA-reverse | TGCATACGCTTTCTCAGCTT |
